# The Role of Orai1 in Regulating Sarcoplasmic Calcium Release, Mitochondrial Morphology and Function in Myostatin Deficient Skeletal Muscle

**DOI:** 10.3389/fphys.2020.601090

**Published:** 2020-12-21

**Authors:** Mónika Sztretye, Zoltán Singlár, Norbert Balogh, Gréta Kis, Péter Szentesi, Ágnes Angyal, Ildikó Balatoni, László Csernoch, Beatrix Dienes

**Affiliations:** ^1^Department of Physiology, Faculty of Medicine, University of Debrecen, Debrecen, Hungary; ^2^Department of Anatomy, Histology and Embryology, Faculty of Medicine, University of Debrecen, Debrecen, Hungary

**Keywords:** SOCE, myostatin deficiency, excitation contraction coupling, mithochondrial defect, mitochondrial calcium uptake

## Abstract

In mice a naturally occurring 12-bp deletion in the myostatin gene is considered responsible for the compact phenotype (Mstn^Cmpt–dl1Abc^, Cmpt) labeled by a tremendous increase in body weight along with signs of muscle weakness, easier fatigability, decreased Orai1 expression and store operated calcium entry (SOCE). Here, on the one hand, Cmpt fibers were reconstructed with venus-Orai1 but this failed to restore SOCE. On the other hand, the endogenous Orai1 was silenced in fibers from wild type C57Bl6 mice which resulted in ∼70% of Orai1 being silenced in whole muscle homogenates as confirmed by Western blot, accompanied by an inhibitory effect on the voltage dependence of SR calcium release that manifested in a slight shift toward more positive potential values. This maneuver completely hampered SOCE. Our observations are consistent with the idea that Orai1 channels are present in distinct pools responsible for either a rapid refilling of the SR terminal cisternae connected to each voltage-activated calcium transient, or a slow SOCE associated with an overall depletion of calcium in the SR lumen. Furthermore, when Cmpt cells were loaded with the mitochondrial membrane potential sensitive dye TMRE, fiber segments with depolarized mitochondria were identified covering on average 26.5 ± 1.5% of the fiber area. These defective areas were located around the neuromuscular junction and displayed significantly smaller calcium transients. The ultrastructural analysis of the Cmpt fibers revealed changes in the mitochondrial morphology. In addition, the mitochondrial calcium uptake during repetitive stimulation was higher in the Cmpt fibers. Our results favor the idea that reduced function and/or expression of SOCE partners (in this study Orai1) and mitochondrial defects could play an important role in muscle weakness and degeneration associated with certain pathologies, perhaps including loss of function of the neuromuscular junction and aging.

## Introduction

Force generation, the main role of skeletal muscle is the result of muscle contraction and relaxation regulated by changes in the intracellular free Ca^2+^ level. The very low resting myoplasmic Ca^2+^ concentration from the nanomolar (nM) range rises rapidly into the micromolar (μM) range through the mechanism termed “excitation-contraction (EC) coupling” during which the action potential triggers Ca^2+^ release from the intracellular store (sarcoplasmic reticulum (SR)) mediated by coupling between the voltage sensor dihydropyridine receptor (DHPR) in the transverse tubule membrane and the ryanodine receptor (RyR1), Ca^2+^ release channel embedded in the SR membrane (Franzini-Armstrong, 2018). Released Ca^2+^ activates the contractile apparatus leading to contraction. During relaxation cytosolic [Ca^2+^] returns to normal level by moving Ca^2+^ back to the SR via SERCA1, the Ca^2+^ pump located mainly in the longitudinal SR.

Depletion of SR calcium stores activate the process called store operated Ca^2+^ entry (SOCE) during which Ca^2+^ enters into the cytoplasm from the extracellular space to replenish the depleted intracellular stores. SOCE is coordinated by two key proteins: stromal interaction molecule 1 (STIM1) and Orai1. The former is the luminal Ca^2+^ level sensor in the SR membrane, while the latter is the Ca^2+^-release activated calcium (CRAC) channel located in the surface membrane (Desai et al., 2015; Vaeth et al., 2017). Deficiency of either molecule impairs SOCE and leads to muscular abnormalities (McCarl et al., 2009; Cully et al., 2012; Li et al., 2012; Zhao et al., 2012; Böhm et al., 2013; Onopiuk et al., 2015; Carrell et al., 2016). The role of SOCE under physiological conditions in muscle is not yet completely determined. Its contribution to muscle development (Kurebayashi and Ogawa, 2001; Wei-Lapierre et al., 2013; Carrell et al., 2016) and myoblast fusion and differentiation (Darbellay et al., 2009) has already been demonstrated, however according to former opinions, because of its slower kinetics, SOCE has not been considered as a relevant process underlying muscle contraction. Recently, an increasing number of studies seem to contradict this idea presenting evidences on the role of SOCE in skeletal muscle contraction, development, fatigue reduction, replenishing SR stores during prolonged muscle contraction (Wei-Lapierre et al., 2013; Carrell et al., 2016; Boncompagni et al., 2017), in maintaining the SR calcium content during EC coupling (Sztretye et al., 2017; Koenig et al., 2018) even under conditions of sustained Ca^2+^ concentration in the SR (Koenig et al., 2019) and disease states (Stiber and Rosenberg, 2011).

Lately, the role of mitochondria in regulating the energy metabolism of muscle cells has been associated to the activity of SOCE (Malli and Graier, 2017) as well. Besides the refill of SR luminal store through SERCA pump, calcium ions can be taken up by mitochondria as well. Although mitochondrial uptake has limited impact on cytosolic [Ca^2+^] regulation (it allots to around 10–18% of the total Ca^2+^ riddance, Yi et al., 2011), it is necessary for energy balance, i.e., to fulfill the high ATP demand of muscle contraction (Porter and Wall, 2012) and its role in governing the intracellular Ca^2+^ signaling in skeletal muscle during EC coupling has also been documented. On the other hand, mitochondrial Ca^2+^ overload can result in dysfunction of mitochondria and degeneration of muscle cells.

Mitochondria can control the ER/SR Ca^2+^ content in many ways and consequently affect the activation, maintenance, and deactivation of SOCE (Parekh, 2008). Also, they are known to have an effect on ER/SR Ca^2+^ release as mitochondrial Ca^2+^ withdrawal from ER/SR Ca^2+^ release channels impacts the degree of ER/SR Ca^2+^ depletion (Malli and Graier, 2017).

In our former studies a mouse strain with a naturally occurring mutation in the myostatin gene [Compact (*Cmpt*); Szabó et al., 1998] has been examined. These mice present a hypermuscular yet reduced muscle-force phenotype accompanied with reduced STIM1 and Orai1 expression, leading to lessened SOCE activity. The contribution of SOCE to the refilling of the SR has also been proven (Sztretye et al., 2017). These observations provided us with an obvious background and paved the rationale for the current study to analyze the role of SOCE partners and potential mitochondrial alterations in calcium release processes and muscle weakness.

Here, the role of Orai1 in the regulation of SR calcium release using either Cmpt muscle fibers reconstructed with venus-Orai1 and wild type fibers with silenced Orai1 expression were studied. Our results favor the idea that Orai1 channels are present in distinct pools responsible for either a rapid refilling of the SR terminal cisternae connected to each voltage-activated calcium transient, or a slow SOCE associated with an overall depletion of calcium in the SR lumen. In this framework, Orai1 channels in the former pool would be permanently associated with STIM partners while those in the latter would have to be recruited into calcium entry units (Protasi et al., 2020) once STIM molecules become activated.

## Materials and Methods

### Animal Care and Experimental Design

Animal experiments conformed to the guidelines of the European Community (86/609/EEC). The experimental protocol was approved by the institutional Animal Care Committee of the University of Debrecen (3-1/2019/DEMAB). The mice were housed in plastic cages with mesh covers and were fed with pelleted standard mouse chow and had access to water *ad libitum*. Room illumination was an automated cycle of 12 h light and 12 h dark, and room temperature was maintained within the range of 22–25°C.

### *In vivo* Electroporation

The mice were anesthetized with 2–4% isoflurane using a V1 Vet Equip tabletop anesthesia machine (Livermore, CA, United States). The O_2_ flow was kept constant at 0.4 L/min. The FDB muscles were injected with 15 μl of 2 mg/ml hyaluronidase in sterile saline. One hour later 20 μg plasmid cDNA of interest was injected at the same location. Ten minutes later, two sterile gold-plated acupuncture needles were placed under the skin on adjacent sides of the muscle. Twenty 100 V/cm, 20-ms square-wave pulses of 1 Hz frequency were applied to the muscle for 1 sec each using an ECM 830, BTX Harvard Apparatus electroporator (Holliston, MA, United States).

### *In vitro* Experiments

Animals were anaesthetized and sacrificed following a protocol approved by the Animal Care Committee of the University of Debrecen (3-1/2019/DEMAB). After CO_2_ overdose and manual cervical dislocation, the *m. flexor digitorum brevis* (FDB) from the hind limb were dissected manually in normal Tyrode’s solution.

#### Isolation of Single Skeletal Muscle Fibers

The manual dissection of FDB muscle in normal Tyrode’s solution was followed by an enzymatic dissociation in the presence of 0.2% Type I collagenase at 37°C for 50 min in a Ca^2+^ free Tyrode’s solution. After the enzymatic treatment FDB muscles were placed in normal Tyrode’s solution and stored in the refrigerator at 4 °C until use for up to 36 h. Single FDB fibers were obtained after gently triturating the muscle with a pipette (Szentesi et al., 1997; Fodor et al., 2008).

All calcium measurements were carried out on single skeletal muscle fibers from the FDB muscles of the mouse. SOCE measurement was performed similarly, as in our earlier report (Sztretye et al., 2017). Briefly, isolated single FDB fibers loaded with the green fluorescent calcium binding dye fluo-8AM (4 μM, 20 min at 20–22°C) were imaged with a laser scanning confocal microscope (5 Live, Carl Zeiss, Oberkochen, Germany) and subjected to multiple manual solution exchanges during which the fluorescence profile versus elapsed time was imaged and then plotted. The dye fluorescence showed a steady state followed by an initial increase (P1), then decrease as a consequence of the application of the releasing cocktail (see below) in a Ca^2+^ –free Tyrode’s medium. This transient corresponded to the depletion of intracellular Ca^2+^ stores via RyR1s. When returning to the 1.8 mM Ca^2+^ in the external solution, another increase should be detected (P2), indicating perhaps SOCE activation and Ca^2+^-influx through surface channels. After the manual delimitation of the cell border, the change of [Ca^2+^]_*i*_ was calculated as ΔF/F_0_, where ΔF is the fluorescence intensity change and was calculated over the cell, whereas F_0_ is the background fluorescence and was calculated in a region next to the cell. The ratio of P2/P1 over time was then calculated and plotted.

#### Voltage Clamp

The experimental design was similar to the one described in our earlier study (Sztretye et al., 2017, 2020). Briefly, isolated FDB fibers were placed in the *external solution* and voltage clamped (Axoclamp 200B, Axon Instruments, Foster City, CA, United States). Changes in cytosolic Ca^2+^ were recorded, following the application of depolarizing voltages and simultaneously imaged using a confocal microscope (Zeiss 5 Live, Oberkochen, Germany). Fibers were dialyzed through the patch pipette with the rhod-2 or fluo-8 and 10 mM EGTA containing *internal solution* and the application of depolarizing pulses was started 15–20 min following the seal formation to assure enough loading time. The ambient temperature was set to 20–22°C. The holding potential was −80 mV and the pipette resistance varied between 2 and 4 MΩ. Correction for linear capacitive currents was performed by analog compensation.

The voltage dependence of activation was described by Boltzmann function:

(1) $$Ca\left(V_{m}\right)=\frac{Ca_{max}}{1+e^{\frac{-\left(V_{m-V_{50}}\right)}{k}}}$$

to derive the transition voltage V_50_ and limiting logarithmic slope 1/k.

Voltage evoked Ca^2+^ transients were analyzed by an in-house custom-made program taking into account the dissociation constant for rhod-2 (K_d rhod–2_ = 1.58 μM). The SR Ca^2+^ content was calculated taking into account the SERCA pump activity, the calcium binding to intracellular buffers as troponin-C, EGTA, parvalbumin and the dye as reported earlier (Sztretye et al., 2017, 2020).

#### Measurement of Mitochondrial Ca^2+^-uptake

Activity-dependent changes in mitochondrial Ca^2+^ levels in single FDB fibers were monitored with rhod-2 as described in Sztretye et al., 2020. FDB fibers were loaded with 5 μM rhod-2-AM for 15 min at room temperature and then washed with dye free normal Tyrode’s solution. Fibers were electrically stimulated (S88 Stimulator, Grass Technologies, Warwick, RI, United States) through a pair of platinum electrodes placed close to the fiber of interest. A single or a series of 5 consecutive tetani (500 ms duration, 100 Hz) were applied at supramaximal activating voltage for each cell. Time series x-y images (512 × 512 pixels, 0.5 ms/pixel) were taken at rest, following the 1st and 5th tetanus. Calculation of rhod-2 fluorescence values originating from the mitochondria (F_mito_) were performed as follows: a line was drawn parallel to the longitudinal axis of the fiber and the fluorescence was calculated at the peaks (I-band fluorescence, representing mitochondria (F_I–band_)) and at troughs (A-band, non-mitochondrial fluorescence, baseline, F_A–band_). Relative mitochondrial Ca^2+^ uptake expressed as F_mito_ was calculated as F_I–band_ – F_A–band_ and then normalized to the baseline fluorescence.

### Confocal Microscopy and Image Analysis

Images of rhod-2, fluo-8, TMRE and Alexa Fluor 488 α-bungarotoxin fluorescence were acquired with a laser scanning confocal microscope (Zeiss 5 Live, Oberkochen, Germany) equipped with a 20x air and a 40x oil objectives. Excitation of rhod-2 was done 543 nm, with emission collected above 550 nm with a long pass filter whereas for fluo-8 the excitation wavelength was 488 nm, the emission was at 520 nm. Line-scan image recordings were synchronized with the application of the depolarizing pulses via pClamp 11.0 (Molecular Devices, CA, United States) and the protocol used is always indicated on the figure. The time resolution was 0.5 ms per line whereas the spatial resolution was 0.24 μm/line. Peak F/F_0_ values were obtained and plotted at close to maximal depolarizations. Single exponential functions were used to fit the time course of the changes of the maximal F/F_0_ values during the series of tetanic depolarizing pulses as described earlier (Sztretye et al., 2017, 2020). Peak F/F_0_ values were then obtained by averaging the data points in the spatial domain and plotted at close to maximal depolarizations. When a series of tetanic depolarizing pulses were applied (200 ms long 8 consecutive pulses at +30 mV) single exponential functions were used to fit the time course of the changes of the maximal F/F_0_:

(2) $$y=y_{0}+ae^{-bx}$$

This then enabled the estimation of SR calcium content as described in our earlier report (Sztretye et al., 2017). In the equation above x is the number of tetanic pulses applied, b is the time constant of SR calcium depletion, a is the remaining calcium in the SR following the last depolarizing pulse and y_0_ is the difference between the total and the remaining SR calcium content following the 8th tetanic pulse.

### Molecular Biology

#### Western Blot

The Western blot technique was applied to detect expression levels of Orai1. Protein samples were prepared from whole FDB muscle homogenates as well as homogenates of isolated single FDB muscle fibers using standard methods of BCA Protein Assay Kit (Thermo Fisher Scientific, Waltham, MA, United States). Protein samples were subjected to SDS-PAGE using 10% precast gels (Bio-Rad Laboratories, Hercules, CA, United States). 20 μg protein per lane was loaded in each case and separated proteins were transferred to 0.2 nitrocellulose membranes (Bio-Rad Laboratories). For immunological detection of Orai1 mouse monoclonal anti-Orai1 (Thermo Fisher Scientific, cat. No. MA5-15777) was used in 1:500 dilution as primary antibody. As for secondary antibody, horseradish peroxidase-conjugated goat anti-mouse IgG (Bio-Rad Laboratories, Hercules, CA, United States, cat. No. 170-6516) was used in a 1:1000 dilution and this was visualized by chemiluminescence reaction (Pierce Biotechnology, Waltham, MA, United States) using LAS-3000 Intelligent Dark Box (Fuji, Tokyo, Japan). For immunolabelling experiments on Cmpt fibers, we marked the endogenous Orai1 protein with Alexa-Fluor Plus 488 secondary antibody (Invitrogen cat. No. A11001) Densitometry of the signals was performed by using ImageJ software (NIH) and optical densities were calculated as normalized to control samples. To prove equal loading, membranes were re-probed with anti–β-actin antibody (Thermo Fisher Scientific, cat. No. MA1-140) or vinculin (Sigma Aldrich, cat. No. V4139) and visualized as described above.

#### Plasmid Preparation

The plasmid venus-Orai1 was kindly provided by R. Dirksen (Univ. of Rochester, NY, United States). The GFP tagged Orai1 shRNA and scrambled shRNA cassettes in pGFP-V-RS vector were purchased from Origene Technologies (cat. No. TG510804). The plasmids were amplified using bacterial cultures grown in the presence of selective antibiotics (kanamycin and ampicillin, respectively). Plasmid DNA preparations were obtained using Endofree Plasmid Kits (Qiagen Inc).

### Electron-Microscopy

FDB muscles were fixed *in situ* with fixative solution (3% glutaraldehyde in Millonig’s buffer) (Sztretye et al., 2017). Small bundles of fixed muscle fibers were then post fixed in 1% OsO4 in water. For rapid dehydration of the specimens, graded ethanol followed by propylene-oxide intermediate was used. Samples were then embedded in Durcupan epoxy resin (Sigma-Aldrich). Ultrathin sections were cut using a Leica Ultracut UCT (Leica Microsystems, Wien, Austria) ultra-microtome and stained with uranyl acetate and lead citrate. Sections were examined with a JEM1010 transmission electron microscope (JEOL, Tokyo, Japan) equipped with an Olympus camera.

### Experimental Solutions

*Kreb’s solution* (in mM): 135 NaCl, 5 KCl, 2.5 CaCl_2_, 1 MgSO_4_, 10 Hepes, 10 glucose, 10 NaHCO_3_; pH 7.2.

*Normal Tyrode’s solution* (in mM): 137 NaCl, 5.4 KCl, 1.8 mM CaCl_2_, 0.5 MgCl_2_, 11.8 HEPES; 1 gL^–1^ glucose; pH 7.4.

*Ca^2+^ free Tyrode’s solution:* same as above except 5 mM EGTA was added and no CaCl_2_ was present.

The “*cocktail”* consisted of 0.4mM 4-CmC, 0.004mM thapsigargin, and 0.05 mM N-benzyl-p-toluene sulphonamide.

*External solution* (in mM): 140 TEA-CH_3_SO_3_, 1 CaCl_2_, 3.5 MgCl_2_, 10 Hepes, 1 4-AP, 0.5 CdCl_2_, 0.3 LaCl_3_, 0.001 TTX (citrate), and 0.05 BTS (N-benzyl-p-toluene sulphonamide). pH was adjusted to 7.2 with TEA-OH and osmolality was adjusted to 320 mOsm with TEA methanesulfonate.

*Internal solution* (in mM): 110 N-methylglucamine, 110 L-glutamic acid, 10 EGTA, 10 Tris, 10 glucose, 5 Na_2_ ATP, 5 PC Tris, 0.5 rhod-2, 3.56 CaCl_2_, and 7.4 mM MgCl_2_ were added for a nominal 1 mM [Mg^2+^] and 100 nM [Ca^2+^]. pH was set to 7.2 with NaOH and osmolality to 320 mOsm with N-methylglucamine.

Fluorescent dyes (rhod-2 tripotassium salt and AM, TMRE, α-bungarotoxin, Alexa Fluor 488 conjugate) were purchased from Invitrogen (Thermo Fischer, Waltham, MA, United States; cat. no. R14220; R1244; T669; B13422). Fluo-8 tripotassium salt was from AAT Bioquest (cat. no. 21087). All other chemicals were purchased from Sigma-Aldrich (St. Luise, MO, United States).

### Statistical Analysis

Pooled data were expressed and plotted as mean ± standard error of the mean (SEM). Student’s two-tailed *t*-test for two independent populations was used for calculating statistical significance (*p* < 0.05).

## Results

### Functional Consequences of Manipulating Orai1 Protein Level by *in vivo* Plasmid Electroporation

**FIGURE 1:**
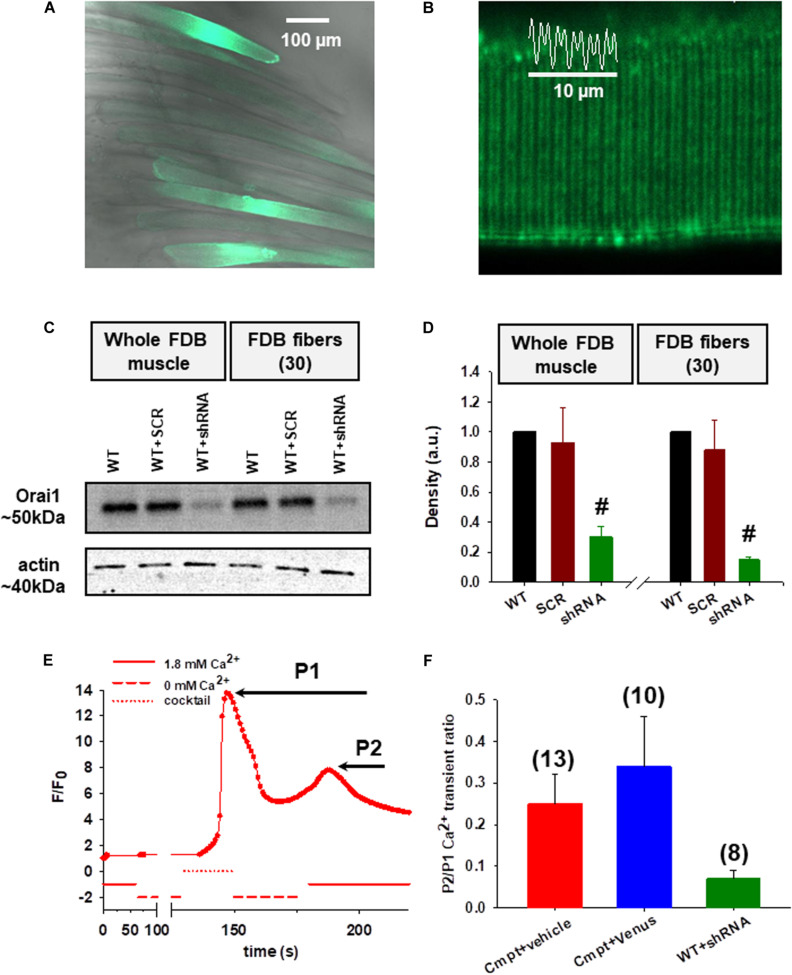
Functional consequences of Orai1 protein level manipulation by *in vivo* plasmid electroporation. **(A)** Good efficiency of venus-Orai1 expression in Cmpt mice where a high percentage of the muscle fibers were transfected as illustrated by them displaying bright fluorescence signals. **(B)** Representative image showing correct targeting of venus-Orai1 to the triad in Cmpt mice as highlighted by the double-banded pattern of venus’ fluorescence. The white trace illustrates the fluorescence intensity profile plotted as a function of distance along the white line for five successive triads. **(C)** Immunoblot analysis in WT mice 5-days after the small-hairpin RNA (shRNA) against Orai1 was introduced into FDB muscles using electroporation showed a significant reduction in the Orai1 protein levels. A scrambled non targeted shRNA probe was used as control along with samples collected from the untreated WT mice. The analysis was performed on whole FDB muscles and also using ∼30 individual FDB fibers identified via the GFP signal and manually collected using a fluorescence microscope. Actin was used as internal control. **(D)** Quantitative densitometry analysis of four independent immunoblots revealed a significant decrease of Orai1 levels compared to WT taken as 100% in both whole FDB muscles and single fibers, respectively (^#^*p* < 0.001) **(E)** A representative SOCE measurement showing the fluorescence profile versus elapsed time following various solution exchanges performed on a Cmpt fiber injected with physiological saline solution (vehicle) and stained with rhod-2 AM. WT FDB fibers were silenced with shRNA-Orai1 5 days before dissection. Following the removal of calcium from the external solution the application of a releasing cocktail (see Materials and Methods and also Sztretye et al., 2017) caused a rapid and robust increase in fluorescence (P1) due to Ca^2+^ release from the SR via RyR1s. After returning to the 1.8 mM Ca^2+^ concentration in the external solution, a slowly activated secondary increase in fluorescence was detected corresponding to the consequent SOCE activation (P2). **(F)** The ratios of the peak of the slow Ca^2+^ transient (P2), representing the activation of SOCE, and that of the depleting-cocktail induced fast SR Ca^2+^ transient (P1). The numbers in parentheses indicate the numbers of individual cells studied. Note that reconstructing the Cmpt fibers with Orai1 does not restore SOCE, but silencing Orai1 in WT fibers does impede proper SOCE function.

In our earlier work (Sztretye et al., 2017) we have shown that endogenous Orai1 levels were reduced in the Cmpt FDB muscles which had direct functional consequences on SOCE activity. Here we decided to reconstruct the Cmpt muscles with venus-Orai1 via *in vivo* electroporation. Five days after the procedure the rate of transfection efficiency was confirmed in FDB fibers by the green fluorescence of the venus-Orai construct (Figure 1A). We observed appropriate targeting of venus-Orai1 to the triad in Cmpt mice as supported by the fluorescence intensity profile plotted as a function of distance (Figure 1B). The correct targeting of the probe is further demonstrated by the representative immunolabelling image of a Cmpt FDB fiber probed with an Orai1 antibody where a similar double row pattern was observed (Supplementary Figure 1A).

Next, we evaluated the effects of silencing Orai1 in wild type C57Bl7 mice. For this, small-hairpin RNA (shRNA) tagged with GFP against Orai1 was introduced into FDB muscles via electroporation. Whole FDB muscles and ∼30 individual fibers identified via their green fluorescence from four different animals were collected and analyzed by Western blot. The representative immunoblot in Figure 1C shows a significant reduction in the Orai1 protein levels on the 5th day following the procedure both on whole muscle and fibers level. A scrambled non targeted shRNA probe (SCR) was used as control along with samples collected from the untreated WT mice. Figure 1D presents the quantitative densitometry analysis of four independent immunoblots. On average Orai1 silencing achieved ∼70% in the whole FDB muscle samples and ∼85% in single fibers. We also analyzed the extent of venus-Orai1 protein expression in whole FDB muscle homogenates by Western Blot (Supplementary Figures 1B,C). While the monoclonal antibody used in our study was excellent in detecting the endogenous Orai1 protein (∼50 kDa) it failed to reveal a band at the expected molecular weight of the venus-Orai1 fusion protein (∼75 kDa). We must clarify that the predicted molecular weight for Orai1 is 33 kDa, however, the observed value is generally between 50 and 60 kDa. In our hands, the Thermo Fisher monoclonal Orai1 antibody (cat. no. MA5-15777) recognizes the protein at ∼50 kDa. The difference is most likely due to post-translational modification of the protein. Based on the above, the quantification of the extent of venus-Orai1 expression was not carried out. Nevertheless, the endogenous Orai1 expression was essentially identical in the vehicle (physiological saline injected and electroporated) and venus-Orai1 electroporated Cmpt FDB muscles.

Store operated calcium entry measurement was performed as described in detail in our previous report (Sztretye et al., 2017). Figure 1E depicts a representative experiment done on an enzymatically dissociated single FDB fiber from a Cmpt mouse that was injected with 15 μl physiological saline solution (vehicle) and electroporated. First, the fiber loaded with rhod-2 AM was placed in the experimental chamber filled with normal Tyrode’s solution (1.8 mM Ca^2+^), which was exchanged manually to a Ca^2+^-free Tyrode’s solution (0 mM Ca^2+^) while continuously imaging the fiber using a confocal microscope with the appropriate laser line. Application of a cocktail (see Materials and Methods, Experimental solutions section) lead to a rapid increase in dye fluorescence due to Ca^2+^ release via RyR1s from the SR (marked as P1). After washout and following the re-addition of 1.8 mM Ca^2+^ concentration in the external solution, a slowly activated secondary increase in fluorescence was observed after the SR store depletion as a result of Ca^2+^ influx via activated Orai1 channels (marked as P2; see also Figure 1B in Sztretye et al., 2017). This, however, was not the case in WT fibers silenced with shRNA-Orai1 (green, Figure 1F and Supplementary Figure 2B) as demonstrated by the lack of secondary peak P2, an indication that virtually no SOCE in these fibers can be detected (Supplementary Table 1). The pooled data of the peaks for the SOCE-associated Ca^2+^ transient (P2) normalized to RyR1-mediated Ca^2+^ transient induced by the depleting-cocktail (P1) revealed a slight increase of SOCE in Cmpt animals reconstructed with venus-Orai1 (blue, Figure 1F; red, Supplementary Figure 2A). This ratio (0.34 ± 0.12) remains, however, below the values we observed and published earlier for WT fibers (0.63 ± 0.09, black column on Figure 1E from Sztretye et al., 2017), suggesting that re-expression of Orai1 in these cells doesn’t restore SOCE function. On Cmpt mice one leg was always injected with physiological saline (vehicle) and electroporated. The P2/P1 ratio for these cells (0.25 ± 0.07) was not statistically different from the untreated Cmpt cells (0.34 ± 0.07, red column on Figure 1E from Sztretye et al., 2017).

Assuming that the SR of the investigated cells are equally loaded, P1 will give a measure of the magnitude of depletion in the SR. Assuming that SOCE is proportional to the depletion, one expects greater SOCE if the depletion is greater. Therefore, although P2 (which is actually the difference between the peak and the baseline) reflects the actual SOCE in that particular fiber, P2/P1 would normalize the SOCE to SR depletion making comparisons across fibers more appropriate.

### Voltage Activation of the FDB Muscles Following Orai1 Manipulation

**FIGURE 2:**
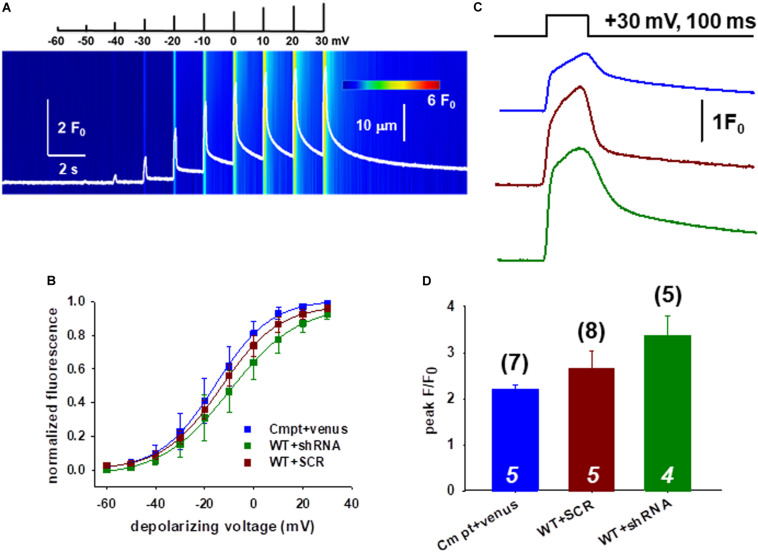
Slight changes of the release channel sensibility to voltage activation in FDB muscles following Orai1 manipulation. **(A)** Representative line-scan image of rhod-2 fluorescence normalized to the baseline value F_0_(x) in a WT fiber expressing the shRNA construct. The cells were held at –80 mV and were subjected to successive depolarizing voltage steps (top) under whole-cell patch-clamp. The pulses lasted 100 ms and the delay between two pulses was 1100 ms. The cells were perfused with 10 mM EGTA via the patch pipette. **(B)** Voltage dependence of the calcium transients. The normalized F/F_0_ values were fitted with a Boltzmann function (see Eq. 1 in section “Materials and Methods”) and then normalized to the obtained maximum. The continuous lines represent the best fit of the Boltzmann function to the average values with the best fit parameters given in Table 1. **(C)** Representative fluorescence intensity profiles plotted for peak F/F_0_ values obtained at maximal individual depolarizing pulses (+30 mV, 100 ms) for Cmpt fibers reconstructed with venus-Orai1 and the WT fibers transfected with SCR and shRNA-Orai1. **(D)** Pooled data for peak F/F_0_ values show no significant changes. The numbers in parenthesis denote the number of individual fibers whereas the numbers in italics denote the number of animals studied.

We continued the functional studies by examining the possibility of altered coupling between the DHPR and RyR1 in single FDB fibers combining whole cell patch clamp and confocal imaging. Figure 2A shows a representative line-scan image of rhod-2 fluorescence from a WT fiber expressing the Orai1-shRNA construct. We have recorded with 10 mV increments calcium transients elicited by a series of 100-ms-long membrane depolarizations in the voltage range between of −60 and +30 mV.

Figure 2B compares the voltage dependence of the normalized maximal fluorescence at the given depolarization from nine WT+SCR (dark red), seven WT+shRNA Orai1 (green) and seven Cmpt fibers expressing the venus-Orai1 construct (blue). The voltage dependence of the normalized fluorescence followed a Boltzmann distribution (Eq. 1 in section “Materials and Methods”); these points were normalized to the obtained maximum for a given fiber and ultimately averaged over the fibers in each group. The solid lines represent the best fit of the Boltzmann function to the average values with the mean values of parameters given in Table 1. The Ca^2+^-release activation in WT silenced fibers was found to be slightly shifted to more negative potentials as underlined by the V_50_ of release, however, this proved to be not different statistically.

**TABLE 1:** The voltage dependence of the normalized fluorescence.

	V_50_ (mV)	k
Cmpt + venus-Orai1 (7)	−15.62 ± 1.55	11.27 ± 0.42
WT + SCR-Orai1 (9)	−13.34 ± 0.75	11.73 ± 0.65
WT + shRNA-Orai1 (7)	−9.89 ± 0.85	13.96 ± 0.75

*V_50_ is the transition voltage and k is the slope of the Boltzmann fit. The mean values of parameters were not significantly different. Numbers in parenthesis are the number of fibers fitted.*

Figure 2C illustrates the temporal profiles of the normalized fluorescence at individual depolarizing pulses with a length of 100 ms measured in single FDB fibers. The average peak F/F_0_ values obtained at maximal depolarizing pulses (+30 mV) are presented in Figure 2D. When comparing the peak F/F_0_ in WT+SCR (dark red) and silenced cells (green) we found slightly increased but statistically unaltered peak values (2.65 ± 0.38 vs. 3.38 ± 0.41) which suggests that the global voltage activated cytosolic Ca^2+^-transients were not affected by the lack of Orai1.

### Fatigability of Individual FDB Fibers Following Orai1 Manipulation

**FIGURE 3:**
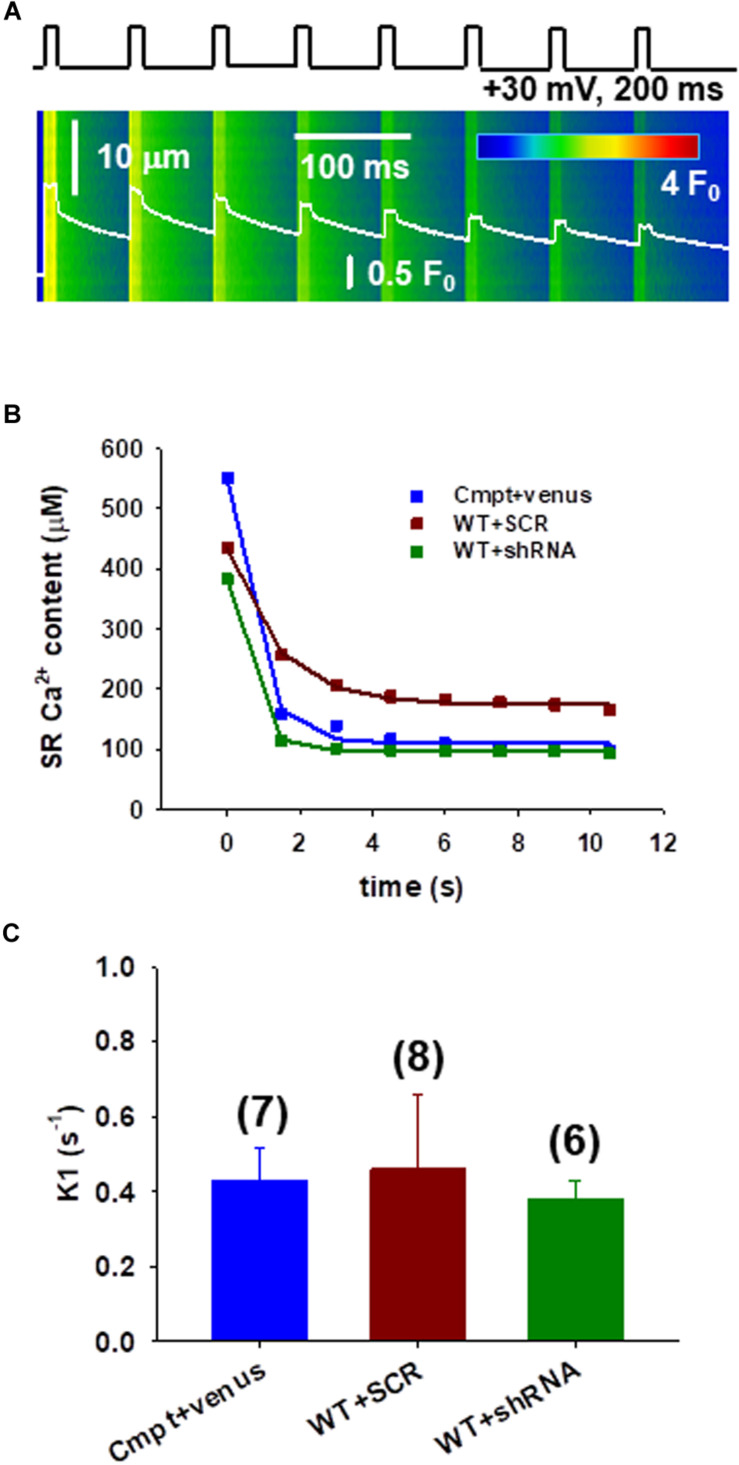
Assessing the calcium fluxes of individual FDB fibers following Orai1 manipulation. **(A)** Line-scan image of the kinetic analysis of Ca^2+^-release fluxes for transients elicited by a train of depolarizing pulses to +30 mV at 1 Hz frequency, lasting 200 ms each in Cmpt FDB fibers reconstructed with venus-Orai1. The white trace depicts the spatially averaged F(t)/F_0_. **(B)** The SR Ca^2+^ content was estimated from the amount released minus the removal flux. A single-exponential function was fitted to the points (for further details please see Eq. A10 in Appendix A from Sztretye et al., 2017). Note that there is a sharp decrease of the SR calcium content following the first depolarizing pulse. **(C)** Pooled data of the calculated fluxes (K1) through Orai1 channels as predicted by our model (see Appendix A from Sztretye et al., 2017). Reconstructing the mutant fibers with venus-Orai1 restored the Ca^2+^ fluxes to the levels previously seen in WT fibers (consult Sztretye et al., 2017, Figure 4F). The numbers in parenthesis denote the number of cells studied.

To assess the calcium fluxes and fatigability in individual FDB fibers under patch clamp a protocol to study the immediate refilling following repetitive stimulation that would also require the correct functioning of SOCE was used. To test this, a series of depolarizations with the arrangement illustrated in Figure 3A were applied. Figure 3B illustrates the change of SR Ca^2+^ content during repetitive stimulation in different fibers. The amount of calcium released decreased by the removal flux was used to calculate the decline in SR Ca^2+^ content, which covers the uptake of Ca^2+^ into the SR by the SERCA pump (Royer et al., 2010; Sztretye et al., 2011, 2017).

Earlier a simple model including three compartments (extracellular, intracellular, and intra-SR spaces (Appendix A in Sztretye et al., 2017) was proposed that is in agreement with an exponential decay in the Ca^2+^ content of the SR under these conditions, where the rate constant of decay (a) is precisely related to the flux (K1) through Orai1 channels. The data points from Figure 3B were thus fitted with exponential functions (Eq.2 in Methods) and K1 values were calculated for each experiment. Figure 3C shows pooled data of K1 (fluxes through Orai1 channels) calculated using our model. The newly acquired data suggests that in the mutant fibers reconstructed with Orai1 (blue) we were able to restore the K1 values to those previously seen in WT (0.34 ± 0.21, black column in Figure 4F from Sztretye et al., 2017) while no significant changes in K1 for WT silenced fibers (green) were detected.

### Activity Dependent Changes in Mitochondrial Calcium Uptake

**FIGURE 4:**
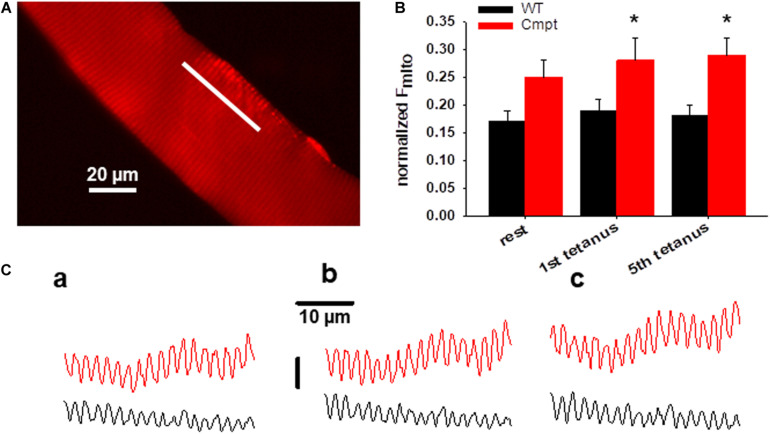
Activity-dependent changes in mitochondrial calcium uptake in single FDB fibers from Cmpt mice. **(A)** Representative image of rhod-2 AM fluorescence in a live Cmpt FDB fiber at rest. **(B)** Average (± SEM) change of normalized mitochondrial fluorescence (F_mito_) calculated as (F_I–band_ – F_A–band_)/F_A–band_ in WT and Cmpt FDB fibers at rest, following 1, and 5 consecutive tetani (*n* = 8 cells, *N* = 3 mice). Note the tendency of mitochondrial calcium accumulation and the higher starting values of the normalized F_mito_ observed in Cmpt. **(C)** Mitochondrial fluorescence measured along the line arbitrarily drawn in parallel with the longitudinal axis of the WT (black) and Cmpt (red) fiber at rest **(a)**, following the first **(b)**, and the fifth consecutive tetanus **(c)**. * denotes significant difference between WT and Cmpt at *p* < 0.05.

Figure 4A presents a representative confocal image of rhod-2 fluorescence on a Cmpt FDB muscle fiber under resting condition. The method of measuring mitochondrial Ca^2+^-uptake has been described in detail in our recent report (Sztretye et al., 2020). In brief, the average normalized fluorescence of mitochondria (F_mito_) calculated as (F_I–band_ – F_A–band_)/F_A–band_ was compared in WT and Cmpt FDB fibers at rest and following tetanic stimulation. The fluorescence was averaged over the spatial domain along a line (white trace) placed in parallel with the fiber’s orientation. Typical traces are shown in Figure 4C. Images were recorded at rest (Figure 4C, panel c1), after the first (c2), and the fifth (c3) consecutive tetanic depolarizing pulses with supramaximal amplitude through platinum electrodes placed in the proximity of the cell studied. The analysis revealed that on average F_mito_ values were already higher at rest in the mutant when compared to WT (0.25 ± 0.03 vs 0.17 ± 0.02, *p* > 0.05) and significantly increased following the first and the fifth tetanic stimulation (0.28 ± 0.03 and 0.29 ± 0.04 in Cmpt vs 0.19 ± 0.02 and 0.18 ± 0.02 in WT, respectively, *p* < 0.05). The pooled data (Figure 4B) clearly indicate the activity dependent mitochondrial calcium uptake as being more pronounced in the mutant.

### Structural Defects of Mitochondria in Cmpt Fibers

**FIGURE 5:**
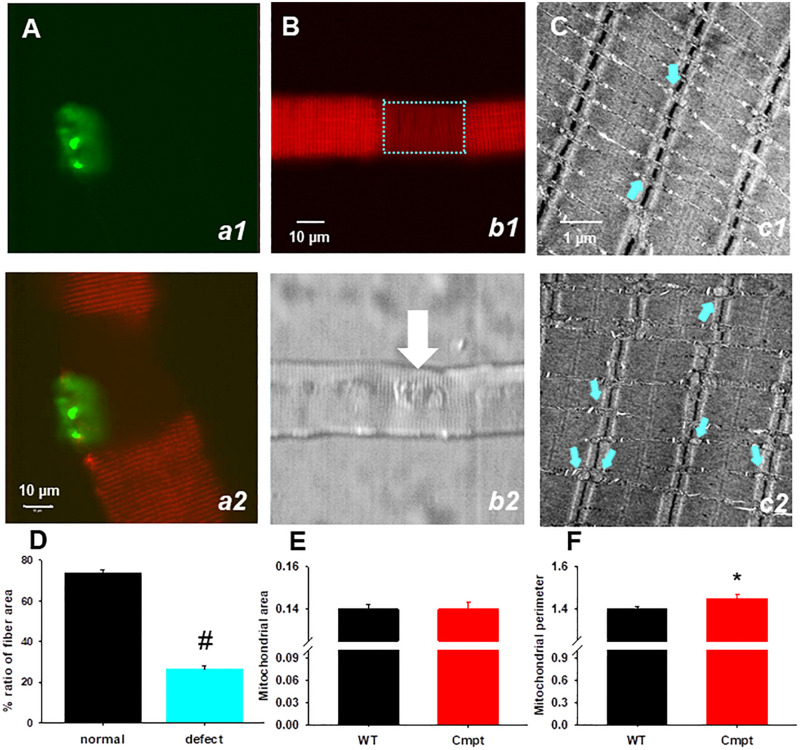
Structural defects of mitochondria in Cmpt fibers. **(A)** FDB live muscle fiber probed with α-bungarotoxin (*panel a1).* Overlay of TMRE (*red*) and α-bungarotoxin images (*green*) of dually stained FDB fiber (*panel a2*). Note that the fiber segment with depolarized mitochondria is overlapped with the NMJ area. **(B)** A segment with depolarized mitochondria is identified in the Cmpt fiber (*panel b1 bracket*) with TMRE staining. The area of mitochondrial defect had variable sizes, but always faced the NMJ (arrow) as suggested by the transmitted light image (*panel b2*) **(C)** Representative electron microscopy images of FDB muscles from 4-month-old WT (*panel c1*) and Cmpt (*panel c2*) fibers. In the WT, mitochondria (*arrows*) aligned within the sarcomeric I band and had uniform sizes. In contrast, Cmpt muscles had enlarged mitochondria (arrows, *panel c2*). Horizontal field width was 4.9 μm, magnification was 30.000x. **(D)** Ratio of fiber area displaying mitochondrial defects (*n* = 28 fiber, *N* = 4 mice). # denotes significant difference at *p* < 0.001. **(E)** Mitochondrial area **(E)** and perimeter **(F)** analysis of electron microscopy images similar to those as presented in C revealed no changes in the area but significant increase in perimeter (*n* = 551 vs. 635, *p < 0.05).

To investigate the mitochondrial morphology in a living muscle cell, FDB fibers from Cmpt mice were incubated with the voltage-sensitive fluorescent indicator TMRE indicating the mitochondrial transmembrane potential, ΔΨ. The distribution patterns of TMRE in the Cmpt muscle fiber displayed localized structural faults, as shown by the regions lacking TMRE staining (Figure 5B, panel b1). Out of 61 cells studied 17 showed defective zones, that is 28%. The areas of mitochondrial defects had uneven sizes but were always localized around the NMJ (Figure 5B, panel b2). The defective areas as revealed by TMRE staining occupied on average 26% of the fiber area (Figure 5D). To determine whether the observed mitochondrial defects in Cmpt fibers were associated with the NMJ, the mitochondrial lesion and the NMJ were imaged simultaneously by staining living Cmpt muscle fibers with TMRE (Figure 5B, b1) and Alexa Fluor 488 conjugated α-bungarotoxin (Figure 5A, a1), a ligand binding to the nicotinic acetylcholine receptors in postsynaptic membranes. Even if the size of area with depolarized mitochondria varied among fibers, this always included the muscle side of the NMJ (Figure 5A panel b2, white arrow). This phenotype was never found in the age-matched WT mice.

Individual FDB muscles isolated from WT and Cmpt mice were used for structural studies and explored in electronmicroscopy images. Figure 5C panel c1 shows the normal arrangement of triads flanked by mitochondria aligned within the sarcomeric I-band having uniform sizes (indicated by cyan arrows). In cross-section images from the Cmpt muscles (panel c2) enlarged mitochondria were identified invading the sarcomeric A-band (cyan arrows). The analysis of these structures revealed significantly increased perimeter (1.40 ± 0.01 vs 1.45 ± 0.003, *p* < 0.05, for WT and Cmpt, respectively, *n* = 635 vs. *n* = 551; Figure 5F) but no differences in the area of the organelles (Figure 5E).

### Functional Defects of Mitochondria in Cmpt Fibers

**FIGURE 6:**
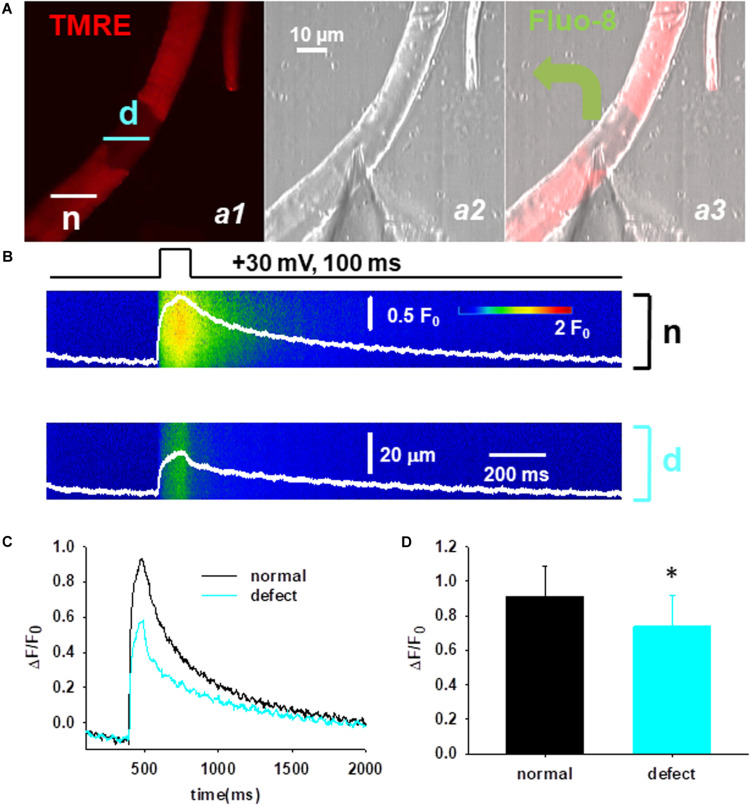
Functional defects of mitochondria in Cmpt fibers. **(A)** Cmpt fibers were first loaded with TMRE to identify the fiber segment with depolarized mitochondria (*panel a1*). n denotes the fiber segment displaying normal mitochondrial membrane potential; d depicts the fiber segment with depolarized or defective mitochondria. The patch pipette was placed at the interface of the regions (*panel a2*). Fluo-8 diffused into the fiber through the pipette (*panel a3*). The pipette solution contained 10 mM EGTA. **(B)** Simultaneous imaging of Ca^2+^ transients of fluo-8 fluorescence in the normal (n) and defective (d) areas during a depolarizing pulse (top). **(C)** Ca^2+^ transients reported by normalized fluo-8 fluorescence changes (*F*/*F*_0_), in the region with normal mitochondria (*black)*, and in the region with depolarized mitochondria (*cyan*). Note the depressed Ca^2+^ transients in the area with defective mitochondria. **(D)** Averaged Δ*F*/*F*_0_ in the fiber region with or without defective mitochondria (*n* = 10 cells, *N* = 3 mice) ^∗^*p* < 0.05.

Lastly, the functional consequences of the mitochondrial defects were studied applying in parallel confocal live cell imaging and the voltage-clamp technique. First, the cells were loaded with TMRE to identify the fiber segments with depolarized mitochondria (Figure 6A, panel a1) and then the cytosolic Ca^2+^ was simultaneously recorded in the normal (n) and defective (d) area (Figure 6B). Figure 6A panel a3 illustrates a representative image of a voltage-clamped FDB fiber that was loaded with the Ca^2+^-sensitive dye fluo-8 AM through the glass pipette allowing the precise recording of Ca^2+^ transients in the cytosol. Line-scan images of voltage induced calcium transients were recorded in the two regions (Figure 6B) and a comparative analysis of the peak F/F_0_ values was performed. Following the application of relatively brief maximal depolarizing pulses (100 ms, +30 mV) smaller calcium transients were observed in regions displaying loss of mitochondrial membrane potential (Figure 6C). On average the calculated F/F_0_ values from ten cells in the normal and defective areas was 0.92 ± 0.18 vs. 0.74 ± 0.18, *p* < 0.05 (Figure 6D).

## Discussion

The role of SOCE in maintaining contractile function of skeletal muscle during sustained stimulation has been widely studied and its contribution to SR refilling required for maintained calcium release during prolonged activity, and to fatigue resistance is generally accepted (Allen et al., 2008; Wei-Lapierre et al., 2013; Pan et al., 2014; Boncompagni et al., 2017). In muscle, this type of SOCE occurs with rather slow kinetics (>1 s timescale), and highly depends on the decline of the SR calcium content. Our laboratory has shown earlier that SOCE plays role in maintaining and refilling the intracellular Ca^2+^ stores, not only during repetitive pronlonged tetanic stimulation, but also following fast activation (Sztretye et al., 2017). Recently, a so-called *phasic* mode of SOCE has been demonstrated directly in skeletal muscle fibers, which is activated under physiological conditions, and this rapid activation of SOCE occurs at a millisecond timescale during each action potential (Koenig et al., 2018), requiring just a local depletion of Ca^2+^ in the SR terminal cisternae.

Orai1 is plentifully expressed in neonatal myotubes and mature muscle fibers as well (Stiber et al., 2008; Dirksen, 2009); it is necessary for the activation of SOCE in myotubes since the elimination of calcium influx through Orai1eradicate SOCE (Lyfenko and Dirksen, 2008; Wei-Lapierre et al., 2013). During the Ca^2+^ depletion of the SR endogenous Orai1 colocalizes with STIM1 or STIM1L at the triad junction to form STIM1/1L–Orai1 complexes making the activation of SOCE possible (Darbellay et al., 2011; Pan et al., 2014; Saüc et al., 2015). The colocalization of STIM1L and Orai1 might be a permanent physical pre-formation in the triad, a configuration which is independent of the SR Ca^2+^ content (Edwards et al., 2010; Darbellay et al., 2011; Koenig et al., 2018).

In this work muscle fibers of myostatin deficient Cmpt mice, where reduced expression of both STIM1 and Orai1 proteins has been previously observed, accompanied with lower SOCE activity, SR content and depolarization induced Ca^2+^ release (Sztretye et al., 2017) were examined.

The results presented here on the effects of manipulation of Orai1 expression in skeletal muscle fibers on SOCE activity might seem contradictory at first sight. The reconstruction of Orai1 with venus-Orai1 via *in vivo* electroporation in Cmpt fibers resulted in an expression level similar to wild type. Nevertheless, SOCE activity at a long timescale has not been affected by the increased levels of Orai1 in reconstructed fibers. The applied protocol for pharmacological depletion of SR did not reveal any significant difference between the magnitudes of the induced, slowly activated SOCE. The depolarization-induced calcium transients were also unaltered. At the same time, in the fibers where endogenous Orai1 has been silenced with shRNA, this type of SOCE has been hindered whereas the peak F/F_0_ of calcium transients evoked by membrane depolarization were not statistically different from WT. These latter observations are in accordance with data published on isolated fibers from inducible, muscle-specific Orai1 knockout mice (Carrell et al., 2016), where this mode of SOCE was absent. That is, the acute overexpression of Orai1 does not lead to assembly of functional STIM1/Orai1 complexes in Cmpt fibers.

On the other hand, reconstructing the Cmpt fibers with venus-Orai1 resettle the Ca^2+^ fluxes through Orai1 channels (more precisely, through the surface membrane Ca^2+^ channels, which we believe to be represented by Orai1 channels) to the levels measured in WT fibers, while in fibers, where Orai1 has been silenced, no significant alterations of Ca^2+^ fluxes have been detected. This phenomenon could be explained by the assumption of two pools of Orai1 channels (as in case of STIM1, however, no structural differences are presumed). One pool is located within the triad in a pre-assembled formation with STIM1L and is responsible for the rapid activation of SOCE in the nanodomain of the junctional membrane, adjacent to the RyR1s, during an action potential (*phasic* SOCE). Because of their occurrence in pairs with STIM1, we refer to this pool as “mated” Orai1 channels. The other pool is distributed along the t-tubule membrane randomly (not necessarily in the transverse t-tubules, where phasic SOCE takes place (Cully et al., 2017), but in longitudinal t-tubules as well). These channels are supposed to be devoid of STIM1 partners in a preset manner, thus we use the term “solitary” for these Orai1 channels. In case of different turnover of these two sets of Orai1 channels (for example because of the limited access to them in the crowded triadic junction, or the diverse composition of the neighboring membrane compartments), the manipulations targeting changes in Orai1 expression could have different impact on them. Our observations imply that electroporation of the venus-Orai1 in the Cmpt muscles has concerned mainly the “mated” pool, since the slowly activating SOCE activity induced by SR depletion in which the “solitary” Orai1 pool is involved remains essentially unaltered. That is, further functional STIM1/Orai1 complexes has not been formed via the slow rearrangement of “solitary” Orai1 to couple with STIM1, while the Ca^2+^ fluxes through the Orai1 channels (estimated by the K1 parameter) increased significantly as compared to untreated Cmpt fibers, denoting an increased number of “mated” Orai1 channels. Acute depletion of Orai1 channels in wild type muscles eventuates in a different response, indicating that the “solitary” pool of Orai1 has been concerned. In these fibers slow SOCE has been abolished almost completely which might reflect to a reduced “solitary” Orai1 pool, however, the K1 parameter has been found to be similar as in WT, that is, the “mated” Orai1 pool has been untouched.

Mitochondria are known to contribute to the activity of SOCE, thus having many roles in the activation, maintenance, and termination of this Ca^2+^ entry pathway (Parekh and Putney, 2005; Parekh, 2008; Demaurex et al., 2009). More recent data by Deak et al., 2014 clearly confirmed the importance of mitochondrial Ca^2+^ for proper SOCE activity. Although the mechanism termed *trans*-mitochondrial Ca^2+^ flux seems very important in regulating SOCE (Malli et al., 2003; Feldman et al., 2010), the precise molecular mechanisms by which mitochondria controls SOCE are still unclear (Malli and Graier, 2017).

At least two different but interrelated molecular mechanisms have been proposed to account for this interaction: i) mitochondria support SOCE activity through their ability to buffer and release Ca^2+^ (Abramov et al., 2007; Parekh, 2008; Demaurex et al., 2009), and ii) mitochondria control SOCE via small metabolites such as pyruvate or ATP, which can either assist the SR Ca^2+^ refilling or prevent the Ca^2+^-dependent inactivation of I_CRAC_ (Montalvo et al., 2006; Demaurex et al., 2009). Although this study did not encompass the above mentioned second mechanism, the analysis of the activity-dependent calcium uptake of the mitochondrial network presented here suggests that the former mechanism in Cmpt fibers is not compromised; rather it is enhanced as underlined by our calcium uptake experiments (Figure 4). This could, partially, explain the lower global cytosolic calcium transients seen in Cmpt mice (see Figure 2D black and red columns).

Increased mitochondrial calcium uptake would then accelerate mitochondrial metabolism resulting in reactive oxygen species (ROS) production (Hempel and Trebak, 2017). While the low level of ROS in the mitochondria acts as an essential signaling molecule regulating several physiological process (D’Autréaux and Toledano, 2007), long term and uncontrolled production of ROS can place the cell under oxidative stress. In skeletal muscle, however, the anomalous ROS production is considered to initiate an intriguing interplay between ROS and Ca^2+^ signaling (Cully and Rodney, 2020). ROS promotes the release of Ca^2+^ from the SR via RyR1 (Marks et al., 2009). The released Ca^2+^ might further amplify the rate of ROS production, establishing a positive feed-back loop of Ca^2+^ and ROS signals leading finally, to disrupted calcium signaling. On the other hand, lower ROS production is associated with decreased RyR1 activity which again manifests in smaller depolarization-induced Ca^2+^ transients.

Altered mitochondrial dynamics are also connected to various neurodegenerative disorders (Zhou et al., 2019). Improper mitochondrial activity participates in neuromuscular relapse in a mouse model of amyotrophic lateral sclerosis (ALS) (Zhou et al., 2010). Mitochondria at the NMJ face display elevated local cytosolic [Ca^2+^] and thereby become depolarized (Zhou et al., 2010). The elevated cytosolic [Ca^2+^] may also decrease mitochondrial motility and promote mitochondrial fragmentation (Yi et al., 2004; Saotome et al., 2008), which could dampen the recovering capacity of damaged mitochondria. In contrast to our current observations on Cmpt mice, the reduced mitochondrial Ca^2+^ uptake led to cytosolic Ca^2+^ hyperactivity at the site of NMJ in muscle fibers of the G93A mouse model of ALS (Yi et al., 2011). The authors explained that the increased cytosolic amplitude of depolarization-induced Ca^2+^ transients could indicate reduced Ca^2+^ capacity of the depolarized mitochondrial region; hence the reduced Ca^2+^ uptake of the mitochondria led to hyperactive cytosolic Ca^2+^ transients.

Interestingly, it was shown that mitofusin 2 (an ER to mitochondria tethering protein) (De Brito and Scorrano, 2008; Ainbinder et al., 2015), corrupts STIM1 trafficking upon ER Ca^2+^ depletion independently from mitochondrial Ca^2+^ handling when mitochondria are completely depolarized (Singaravelu et al., 2011). Filadi et al. (2015) showed that cells lacking mitofusin 2 have decreased expression levels of the mitochondrial calcium uniporter (MCU) which could explain the reduced mitochondrial Ca^2+^ signals they encountered. Albeit the role of mitofusin 2 and MCU were not explored in our study, this could be a possible explanation for the decrease in the amplitude of the calcium transients in the areas presenting local defects in mitochondrial ΔΨ. Another possibility that would explain this phenomenon could be due to alterations in ROS generation in the defective cell regions.

## Data Availability Statement

The raw data supporting the conclusions of this article will be made available by the authors, without undue reservation.

## Ethics Statement

The animal study was reviewed and approved by Animal Care Committee of the University of Debrecen.

## Author Contributions

MS, BD, IB, PS, and LC wrote the manuscript, contributed to discussion and reviewed and edited the manuscript. MS, ZS, and BD did *in vitro* calcium experiments. NB did the Western Blot experiments. GK did the electron microscopy. MS, PS, ZS, ÁA, and NB analyzed the data. MS and PS did the statistical analysis. All authors contributed to discussion, laboratory support, and reviewed/edited the manuscript.

## Conflict of Interest

The authors declare that the research was conducted in the absence of any commercial or financial relationships that could be construed as a potential conflict of interest.

## Supplementary Material

Supplementary Figure 1 **(A)** Confocal image of an isolated Cmpt FDB fiber where endogenous Orai1 was labeled with an Alexa-Fluor-488 conjugated antibody. **(B)** Western blot analysis performed on whole FDB muscle homogenates obtained from two Cmpt mice injected with either vehicle or the venus-Orai1 construct. Vinculin was used as an internal control. **(C)** Densitometric analysis of the gel presented in panel B. Note that we were unable to detect an increase in the expression at the molecular weight where Orai1 protein is expected in control animals (∼50 kDa). In fact, we were unable to detect any band at the predicted molecular weight of the venus-Orai1 fusion protein (∼75 kDa). We can only reconcile these results if we assume that our monoclonal antibody is unable to recognize the venus-Orai1 fusion protein due to modification of the epitope that the antibody recognizes.

Supplementary Figure 2 Representative SOCE measurements carried out on **(A)** Cmpt+venus-Orai1 and **(B)** WT+shRNA electroporated FDB fibers, respectively. Experimental protocol was identical as presented in Figure 1E.

Supplementary Table 1 The compiled list of individual cells examined during the SOCE measurements. P1: cocktail induced SR calcium transient; P2: SR depletion activated SOCE transient. Highlighted data correspond to the traces presented in Figure 1E and Supplementary Figures 2A,B.
