# Supplementary figures and images for: The Role of Orai1 in Regulating Sarcoplasmic Calcium Release, Mitochondrial Morphology and Function in Myostatin Deficient Skeletal Muscle
Source: Front Physiol. 2020 Dec 21;11:601090. doi: 10.3389/fphys.2020.601090 (PMC7779810; doi:10.3389/fphys.2020.601090)

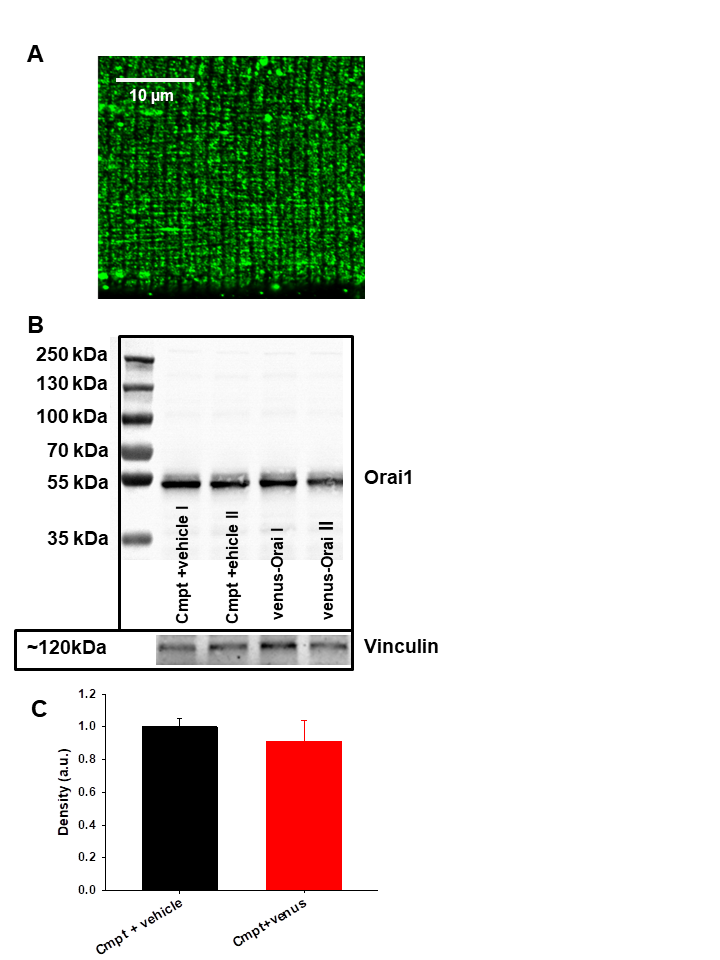

Supplement: Supplementary Figure 1 — (A) Confocal image of an isolated Cmpt FDB fiber where endogenous Orai1 was labeled with an Alexa-Fluor-488 conjugated antibody. (B) Western blot analysis performed on whole FDB muscle homogenates obtained from two Cmpt mice injected with either vehicle or the venus-Orai1 construct. Vinculin was used as an internal control. (C) Densitometric analysis of the gel presented in panel B. Note that we were unable to detect an increase in the expression at the molecular weight where Orai1 protein is expected in control animals (∼50 kDa). In fact, we were unable to detect any band at the predicted molecular weight of the venus-Orai1 fusion protein (∼75 kDa). We can only reconcile these results if we assume that our monoclonal antibody is unable to recognize the venus-Orai1 fusion protein due to modification of the epitope that the antibody recognizes. [file Image_1.TIF]

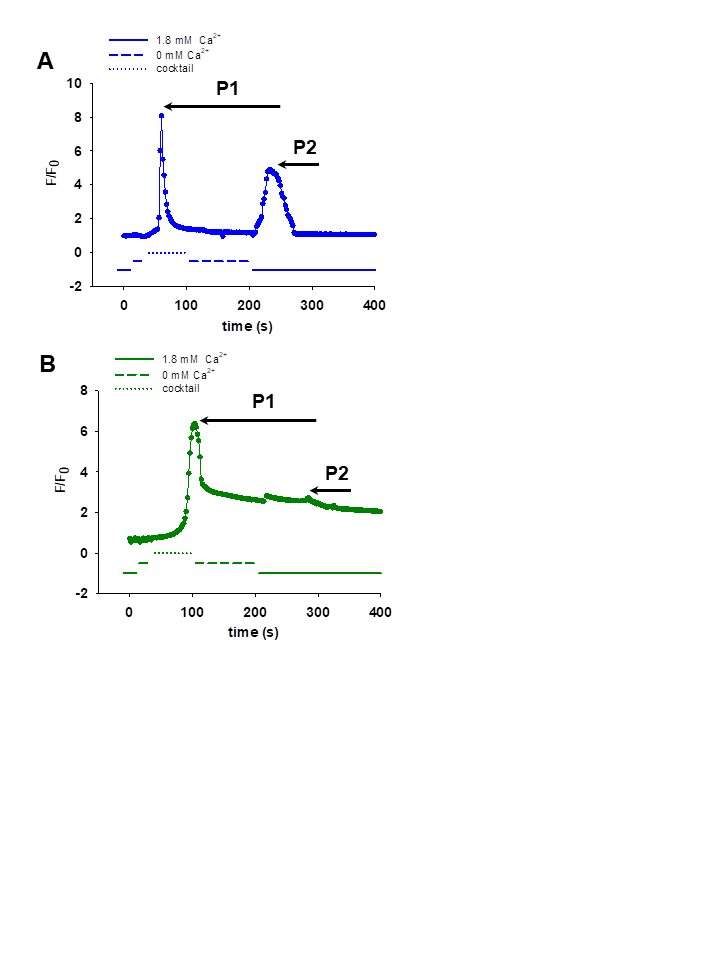

Supplement: Supplementary Figure 2 — Representative SOCE measurements carried out on (A) Cmpt+venus-Orai1 and (B) WT+shRNA electroporated FDB fibers, respectively. Experimental protocol was identical as presented in Figure 1E. [file Image_2.TIF]
