# Supplementary material for: The Role of Orai1 in Regulating Sarcoplasmic Calcium Release, Mitochondrial Morphology and Function in Myostatin Deficient Skeletal Muscle
Source: Front Physiol. 2020 Dec 21;11:601090. doi: 10.3389/fphys.2020.601090 (PMC7779810; doi:10.3389/fphys.2020.601090)
Supplement: Supplementary Table 1 — The compiled list of individual cells examined during the SOCE measurements. P1: cocktail induced SR calcium transient; P2: SR depletion activated SOCE transient. Highlighted data correspond to the traces presented in Figure 1E and Supplementary Figures 2A,B. [file Table_1.DOCX]

| Cmpt+vehicle  Nr. of cells studied | P1 | P2 | P2/P1 |
| --- | --- | --- | --- |
|  |  |  |  |
| 1  2  3  4  5  6  7  8  9  10  **11**  12  13 | 6.65  13.71  5.95  8.01  8.91  6.98  4.82  4.76  13.42  20.02  **12.45**  9.63  7.37 | 0.08  11.82  0.88  1.19  3.81  2.62  0.5  3.92  0.16  1.93  **2.4**  0.2  0.32 | 0.01  0.86  0.14  0.14  0.42  0.37  0.10  0.82  0.01  0.10  **0.19**  0.02  0.04 |
|  |  |  |  |
| **Average**  **SE** | **9.43**  **1.17** | **2.29**  **0.84** | **0.25**  **0.07** |

| Cmpt + venusOrai1  Nr. of cells studied | P1 | P2 | P2/P1 |
| --- | --- | --- | --- |
|  |  |  |  |
| 1  2  3  4  5  6  **7**  8  9  10 | 12.54  4.35  14.24  1.4  2.43  7.72  **7.11**  3.41  3.94  2.86 | 1.66  1.25  1.52  0.88  0.9  0.04  **3.83**  0.04  5.02  0.18 | 0.13  0.29  0.11  0.63  0.37  0.01  **0.54**  0.01  1.27  0.06 |
|  |  |  |  |
| **Average**  **SE** | **6.00**  **0.34** | **3.27**  **1.43** | **0.34**  **0.12** |

| WT + shRNAOrai1  Nr. of cells studied | P1 | P2 | P2/P1 |
| --- | --- | --- | --- |
|  |  |  |  |
| 1  2  3  4  5  6  **7**  8 | 12.27  3.18  5.18  6.96  4.66  6.25  **5.62**  1.07 | 1.64  0.02  0.23  0.49  0.04  0.84  **0.27**  0.09 | 0.13  0.01  0.04  0.07  0.01  0.13  **0.05**  0.08 |
|  |  |  |  |
| **Average**  **SE** | **5.65**  **1.15** | **3.27**  **0.20** | **0.07**  **0.02** |
